# Low back pain risk factors in a large rural Australian Aboriginal community. An opportunity for managing co-morbidities?

**DOI:** 10.1186/1746-1340-13-21

**Published:** 2005-09-30

**Authors:** Dein Vindigni, Bruce F Walker, Jennifer R Jamison, Cliff Da Costa, Lynne Parkinson, Steve Blunden

**Affiliations:** 1 Private practice of chiropractic, 12 David Street, Lalor, Victoria, 3075, Australia; 2 School of Medicine, James Cook University, Townsville, Queensland, Australia; 3 School of Chiropractic, Murdoch University, Western Australia; 4 School of Mathematical & Geospatial Sciences, RMIT University, Melbourne, Australia; 5 Centre for Research and Education in Ageing, Faculty of Health, The University of Newcastle, New South Wales, Australia; 6 Chief Executive Officer, Durri Aboriginal Corporation Medical Service, Kempsey, New South Wales, Australia

**Keywords:** Low back pain, risk factors, chiropractic, general health, Australian, Aboriginal, Indigenous

## Abstract

**Background:**

Low back pain (LBP) is the most prevalent musculo-skeletal condition in rural and remote Australian Aboriginal communities. Smoking, physical inactivity and obesity are also prevalent amongst Indigenous people contributing to lifestyle diseases and concurrently to the high burden of low back pain.

**Objectives:**

This paper aims to examine the association between LBP and modifiable risk factors in a large rural Indigenous community as a basis for informing a musculo-skeletal and related health promotion program.

**Methods:**

A community Advisory Group (CAG) comprising Elders, Aboriginal Health Workers, academics, nurses, a general practitioner and chiropractors assisted in the development of measures to assess self-reported musculo-skeletal conditions including LBP risk factors. The Kempsey survey included a community-based survey administered by Aboriginal Health Workers followed by a clinical assessment conducted by chiropractors.

**Results:**

Age and gender characteristics of this Indigenous sample (n = 189) were comparable to those reported in previous Australian Bureau of Statistics (ABS) studies of the broader Indigenous population. A history of traumatic events was highly prevalent in the community, as were occupational risk factors. Thirty-four percent of participants reported a previous history of LBP. Sporting injuries were associated with multiple musculo-skeletal conditions, including LBP. Those reporting high levels of pain were often overweight or obese and obesity was associated with self-reported low back strain. Common barriers to medical management of LBP included an attitude of being able to cope with pain, poor health, and the lack of affordable and appropriate health care services.

Though many of the modifiable risk factors known to be associated with LBP were highly prevalent in this study, none of these were statistically associated with LBP.

**Conclusion:**

Addressing particular modifiable risk factors associated with LBP such as smoking, physical inactivity and obesity may also present a wider opportunity to prevent and manage the high burden of illness imposed by co-morbidities such as heart disease and type-2 diabetes.

## Background

Low back pain (LBP) is the most prevalent musculo-skeletal condition in rural and remote communities [1-3]. Indigenous people in these communities are over-represented in low-skilled, manual jobs and the community-service sector [4]. As such they are more likely to be exposed to greater manual handling of loads, repetitive strains and risk of musculo-skeletal conditions. Formal reporting of such conditions in the Australian Indigenous community is infrequent [1]. These occupational factors and resulting LBP may be compounded by lifestyle risk factors including smoking, physical inactivity, and obesity [5].

There is an abundance of literature reporting on the risk factors associated with LBP in the general population [6]. Known modifiable risk factors for low back pain are lack of fitness, poor health, obesity, smoking, drug dependence, and occupational factors including heavy lifting, twisting, bending, stooping, awkward posture at work and prolonged sitting. Those that are non-modifiable are increasing age, number of children, a previous episode of LBP and major scoliosis [6]. Within the public health context it is important to prevent injuries and painful conditions by addressing modifiable risk factors [7-9].

Australian Indigenous communities experience sub-optimal mortality and morbidity rates. As such it has been argued that by adopting a holistic approach and addressing modifiable risk factors associated with LBP, such as smoking, physical inactivity and obesity, the clinical management of co-morbidities such as heart disease and diabetes may also be partially addressed [10]. Exercise, for example, has been reported as the single most important lifestyle factor for preventing and managing insulin resistance especially among those who are obese [11,12] It is also known that once their presenting musculoskeletal condition has been effectively managed, patients are more likely to comply with their practitioner's advice to promote other aspects of their health including weight loss and increased physical activity [10].

Modifiable risk factors for LBP mentioned above have been further classified as lifestyle (physical inactivity, poor muscle strength, obesity, smoking), and occupational (heavy lifting, twisting, bending, stooping, prolonged sitting, awkward posture at work, previous history of injury to the area) [6]. These are summarised in Table 1. Where high levels of evidence (Level I evidence) such as meta-analyses or systematic reviews were not available, less rigorous studies (Level II, III and IV evidence) were reported to represent the current levels of knowledge.

**Table 1:** Individual modifiable risk factors associated with low back pain

	**Factors strongly associated with LBP (OR > 1.2-)**	**Factors moderately associated with LBP (OR ≥ 1–1.2-)**
**Lack of fitness/Physical inactivity**	Balague, 1999 [44]* Feuerstein, 1999[45] ****	
**Smoking**	Balague, 1999[44] * Feldmann, 1999[47] *** Levangie, 1999[48] *** Power, 2001[49] **	Leboeuf-Yde, 1995[46] *
**Obesity**	Koda, 1991[50] **** Alcouffe, 1999[51] **** Walker, 1999[52] ** Fransen, 2002[53] **** Webb, 2003[55] ****	Leboeuf-Yde, 1999[46] * Balague, 1999[44] * Levangie, 1999[48] **** Lecerf, 2003[54] **** Mirtz, 2005[56] **
**Psychosocial stress**	Balague, 1995[57] *** Hagg, 1997[58] **** Josephson, 1998[60] **** Adams, 1999[61] *** Krause, 1998[62] *** Feuerstein, 1999[45] **** Bildt, 2000[63] *** Thorbjornsson, 2000[64] *** Vingard, 2000[65] **** Yip, 2001[66] **** Power, 2001[67] ** Harkness, 2003[68] *** Van den Heuvel, 2004[69] ***	Balague, 1999[44] * Hoogendorm, 2000[59] ***
**Physical trauma**	Harkness, 2003[68] *** Balague, 1999[44] *	
	**Factors strongly associated with LBP (OR > 1.2-)**	**Factors moderately associated with LBP (OR > 1–1.2-)**
**Awkward posture (at work)**	Koda, 1991[50] **** Alcouffe, 1999[51] **** Jin, 2000[53] **	Picavet, 2000[70] ***
**Frequent bending and twisting**	Alcouffe, 1999[51] **** Hoogendoorm, 2000[59] *** Vingard, 2000[65] **** Jin, 2000[71] ** Van den Heuvel, 2004[69] ***	Picavet, 2000[70] ***
**Heavy lifting, repetitive lifting**	Suadicani, 1994[72] **** Marras, 1995[73] **** Magnusson, 1996[74] **** Sturmer, 1997[75] **** Krause, 1998[62] *** Josephson, 1998[60] **** Alcouffe, 1999[51] **** Thorbjornsson, 2000[64] *** Vingard, 2000[65] **** Hartvigsen, 2001[76] *** Nahit, 2001[77] **** Fransen, 2002[53] **** Harkness, 2003[68] ***	
**Jarring, Gripping, vibration, repetitive actions**	Bongers, 1993[78] * Magnusson, 1996[74] **** Levangie, 1999[48] *** Pope, 1999[79] *** Jin, 2000[71] *	
**Prolonged sitting & prolonged standing**	Burdorf, 1994[80] **** Bongers, 1993[78] ** Thorbjornsson, 2000[64] ***	Hartvigsen, 2000[81] ***

NB: Only first authors included.

**Legend: **+ OR: Odds ratio

Level I evidence *, Level II evidence **, Level III evidence ***, Level IV evidence****

• Level I – based on studies such as meta-analyses or systematic reviews of all relevant randomised controlled trials (RCTs);

• Level II – based on well-designed RCTs;

• Level III – based on well-designed prospective or case-control analytical studies; and

• Level IV – based on opinions of respected authorities, clinical experience, descriptive studies and case reports or reports of expert committees.

As part of a study investigating the prevalence of LBP in this community [3], the risk factors known to be associated with LBP and other serious causes of morbidity and mortality were measured. This paper aims to describe the most commonly reported risk factors for LBP in a large rural Indigenous community; and examine their association with reported LBP as a basis for informing the development of a broad health promotion intervention in this community.

## Methods and materials

### Design

A cross-sectional self-report survey (Kempsey survey) was conducted to determine the extent of risk factors (Table 1) and their association with LBP in the study community.

### Ethics: consent and approval

Participating community members completed a consent form that explained the purpose of the survey. Ethics approval was obtained from the Durri Aboriginal Corporation Medical Service (ACMS) Board of Directors and the Human Research Ethics Committee of the University of Newcastle.

### Community consultation, collaboration and ownership of the program

The Durri Community of Kempsey, NSW, Australia, comprises one of Australia's largest rural Aboriginal communities. The Durri (ACMS) is at the forefront of providing culturally appropriate care, largely via its Aboriginal Health Workers (AHWs). Durri ACMS aims to:

'make primary health care and education accessible to all members of the community in a culturally appropriate and spiritually sensitive manner, endeavouring to improve not only the health status but also the well-being of the Durri Aboriginal community' [13].

Discussions with a cross section of community members led to the formation of a Community Advisory Group (CAG) (which included representatives from the Durri ACMS, Booroongen Djugun Aboriginal Health Worker College, Hands On Health Australia and the University of Newcastle). The CAG aimed to advise on the development and implementation of the musculo-skeletal prevalence study [14]. Aboriginal Health Workers were chosen as the study agents because they are recognised as essential in providing culturally appropriate and effective health-care for their communities [15-22].

Community consultation occurred throughout the study. This process involved regular discussions with key-informants from the community including AHWs, elders and health professionals. The community was informed of developments via information sheets and the publication of a summary report during the process and at the completion of the study.

### Sample

Our goal was to select a representative cross-sectional sample of the local Aboriginal community of sufficient size to generalise our major findings to the whole local community (population 550). A random sampling procedure stratifying for age and sex was used to derive a representative sample of the local community. The sample size was generated using Epi-Info 6 [23]. With a population size of 550, the expected frequency of the main variable of interest (low back pain) was estimated at 50%. The value chosen as the farthest acceptable from the real population was 44%. Using these values and a 95% confidence interval, the ideal random sample size calculated was 180. However, we expected that logistically this was unlikely to be achieved, as many of the sample selected were likely to be uncontactable given the transient nature of community residents [24]. Accordingly, where randomly selected community members were unable to participate, they were replaced using a convenience sampling approach to achieve the required sample size. Although this strategy was not ideal, all attempts were made to attain a representative sample. Participants within the community were selected from persons aged 15-years or older who had been previously identified as Aboriginal (according to the definition of Aboriginal adopted by the Department of Aboriginal Affairs Constitutional Section) [25]. These participants were recruited by distributing letters inviting them to contact the assisting AHWs at the ACMS. If no response was received within a week, an attempt to contact the person via telephone was made by the assisting AHW.

### Procedure

The Kempsey survey included a screening survey administered by Aboriginal Health Workers immediately followed by a clinical conducted by chiropractors blinded to the findings of the screening survey.

Those who consented to participate were asked to attend the Durri ACMS. If participants found transport to the ACMS difficult, either the research team (including the researcher, the AHW and volunteer chiropractors/chiropractic students) would travel to the participants' homes, or the assisting AHW would arrange for the Durri ACMS bus to provide transportation at no charge.

### Screening survey

Participants completed a screening survey previously found to be culturally acceptable and sensitive in measuring musculo-skeletal conditions and associated risk factors in this community. The survey achieved satisfactory measurement agreement (Kappa scores) when compared to a clinical assessment performed by chiropractors (a proxy "Gold Standard") [22]. Although some authors argue that a 'Gold standard' does not exist in many areas of musculo-skeletal practice [26], standard clinical assessments performed by musculo-skeletal health professionals provide the best available tools for measuring painful and limited ranges of motion and a provisional diagnosis [27]. The purpose of the screening survey was to identify those who had experienced a musculo-skeletal condition including ache, pain or discomfort. The questionnaire also assessed self-reported limitations to Activities of Daily Living (ADL) imposed by pain.

Participants screened by the AHW-administered survey subsequently underwent a clinical examination conducted by four chiropractors previously trained and assessed in standard, clinical assessment procedures according to a procedural manual which outlined the cultural considerations and logistical processes required by researchers. The content of the procedural manual was revised in a two-hour workshop for participating researchers to clarify and standardise study requirements. The exam was based on accepted clinical parameters for conducting musculoskeletal conditions and included the domains of assessment used by teaching institutions [28]. Thus attempts were made to fulfil content and face validity.

### Assessment

Participants attended a clinical assessment immediately following the screening survey to confirm the presence of musculo-skeletal conditions [22]. Chiropractors and 5th year chiropractic students performed a follow-up clinical assessment (based on clinical assessment parameters used in 1999 at the School of Chiropractic, RMIT University, Victoria, Australia) [28] to validate the findings reported in the screening questionnaire.

A positive pain finding in the clinical assessment was derived by practitioner-based examination, including the patient's history of involved site(s) followed by standard orthopaedic and range of motion tests to localise sites of pain and restricted movement. A negative pain finding was indicated by the absence of reported pain and/or restricted orthopaedic and range of motion findings as examined by the practitioner. Trivial LBP was differentiated from important LBP using a Likert scale. High levels of pain were interpreted as those ranging between 6–10 on a Likert scale of 0–10. Only those reporting "High" levels of pain were analysed in this study. Further questions related to any musculo-skeletal condition(s) experienced in the last seven days. In particular, probable causes of symptoms, past history, initial episode(s) of symptoms, duration of symptom(s), 'average' severity of symptoms and any associated limitation of daily activities. Also examined were, social routine and work activities, the type of treatment received and any barriers to receiving treatment were sought.

In the history component of the clinical assessment, chiropractors once again questioned participants about the presence of musculo-skeletal risk factors (according to the criteria reported in Table 1). Risk factor data were derived in the history component of the clinical assessment by asking questions from a list of modifiable occupational and lifestyle factors. Results for LBP as measured in the clinical assessment were used in the analysis. Clinical findings requiring follow-up treatment, management or referral was also identified.

Health workers using a laptop computer entered data on-site into a specifically designed, Microsoft Access database.

### Screening and assessment agreement

The questionnaire results were compared to the data from the clinical examination and published in a previous study (Table 2). Eighty-three percent of all participants reporting LBP in the screening survey also tested positive for LBP via the clinical assessment. Sensitivity of the screening survey for LBP was 0.83, specificity 0.63 and Kappa 0.46. Thus the screening survey achieved an adequate level of agreement with the clinical assessment [29].

**Table 2:** Sensitivity, specificity and Kappa for LBP screening survey compared to clinical assessment (n = 189)

**Survey results**	**Clinical Assessment**					
				
	**Negative**	**Positive**	**Total**	**Sensitivity**	**Specificity**	**Kappa coefficient**
**Negative**	43	21	64	**0.83**	**0.63**	**0.46**
**Positive**	25	100	125			
**Total**	**68**	**121**	**189**			

### Measures

The main variables of interest from the survey and clinical assessment were:

• Demographic and other sample characteristics-age, sex, number of children, occupation, weight, and Body Mass Index (BMI).

• Prevalence of LBP (within the last seven days, according to self report).

• Pain levels were recorded using a Likert scale where a score of 0 corresponded to no pain and 10 to severe pain.

• Duration of LBP was categorised as less than/equal to or more than seven weeks.

• Disability levels were recorded using a Likert scale where a score of 0 corresponded to no disability and 10 to severe disability. Disability was defined as "how much the condition (ache, pain or discomfort) had affected the participants ability to carry out daily activities (e.g., housework, washing, dressing, lifting, walking, driving, climbing stairs, getting in and out of bed or a chair, sleeping, working, social activities and sport)".

• Self-reported modifiable risk factors as described in Table 1 (according to a standardised clinical history).

• Other musculo-skeletal conditions.

### Analyses

Frequencies and confidence intervals were reported for characteristics of the sample, prevalence of LBP and reported risk factors for low back pain. Chi-square analyses were performed to test for factors associated with low back pain. Given the number of variables, only significant associations were reported.

## Results

### Sample

The study was conducted between January 2001 and July 2002. The sample comprised 189 Indigenous people: 80 were selected randomly and the remainder were convenience sampled as described above.

### Sample characteristics

#### Age and sex

The mean age of participants was 44 years ( ± 14.8) and the median age 43 years. The sample comprised 87 males (46%) and 102 females (53%) ranging in age from 15 to 80 years. There were no significant differences in the distribution of males and females in the various age categories (p = 0.35). Gender was comparable with previous ABS census data for Indigenous people in Australia [26]. Age categories were also similar in breakdown to those described in census data for the entire Indigenous community (Table 3) [30].

**Table 3:** Age and sex of study participants

**Age category (years)**	**Male**	**Female**	**Total**	**% Male**	**% Female**	**% Total**
**15 – 25**	20	20	40	23.0	19.6	21.2
**26 – 35**	14	16	30	16.1	15.7	15.9
**36 – 45**	25	29	54	28.7	28.4	28.6
**46 – 55**	13	10	23	14.9	9.8	12.2
**56 +**	12	24	36	13.8	23.5	19.0
**Unknown**	3	3	6	3.4	2.9	3.2

**Total**	**87**	**102**	**189**	**100**	**100**	**100**

Despite a high consent rate (85% of the randomly recruited sample), the response rate was low (40%) because many members of this highly mobile community were unable to be contacted.

#### Number of children

Approximately one third (31%) of participants had between two or three children. Thirty percent of participants had no dependent children and 17% had 4–5 children. Of note, 15% had six or more children. These findings are comparable to those of other Indigenous studies [5]. An Australian Bureau of Statistics (ABS) study reported that Indigenous families tend to be larger than Australian families overall. According to the 1996 Census, approximately 13% of Indigenous families had four or more children compared with less than 5% of other Australian families [5].

#### Occupation

Occupational demographics of the participants in the study are summarised in Table 4. Approximately one third of the community surveyed were students or unemployed. A significant number of people surveyed were associate professionals, retired workers, involved in home duties or labourers. These data were generally comparable with those reported for Indigenous people by the ABS (2000). However, for males in the Kempsey survey, there were significantly less professionals, managers, tradespersons and transport workers, and more intermediate clerical, sales and service persons, compared to the ABS population. For females there were significantly more professional, and associates professionals (such as Aboriginal Health Workers), and less tradespersons or transport workers as well as many less intermediate clerical, sales and service persons, compared to the ABS population [5].

**Table 4:** Occupation of study participants according to sex

**Occupation**	**Male**	**Female**	**Total**	**% Male**	**% Female**	**% Total**
**Managers and Administrators**	5	3	8	5.7	2.9	4.2
**Professionals**	7	9	16	8.0	8.8	8.5
Associate professionals*	5	16	21	5.7	15.7	11.1
**Tradespersons and related workers**	1	2	3	1.1	2.0	1.6
**Advanced clerical and service workers**	3	2	5	3.4	2.0	2.6
**Intermediate clerical, Sales and service workers**	3	2	5	3.4	2.0	2.6
**Elementary Clerical, Sales and Service workers**	2	6	8	2.3	5.9	4.2
**Labourers and Related workers**	13	3	16	14.9	2.9	8.5
**Unemployed/Student**	38	28	66	43.7	27.5	34.9
**Home duties**	1	16	17	1.1	15.7	9.0
**Retired**	4	15	19	4.6	14.7	10.1
**Unknown**		5	0	5	0.0	2.6

**Total**	**87**	**102**	**189**	**100**	**100**	**100**

* Associate Professionals

#### BMI

Table 5 shows that 32% of participants were overweight and 39% were obese. Using Body Mass Index (BMI) estimates, 26% (95% CI: 20%–32%) of participants were overweight (BMI = 25.0–29.9) and 45% (95% CI: 38%–52%) were obese (BMI = 30.00). The high prevalence of obesity in this study agrees with national figures demonstrating a greater prevalence of obesity among Indigenous people than non-Indigenous Australians [5].

**Table 5:** Body Mass Index (BMI) of participants, according to age and sex (n = 189)

**BMI classification**											
**Age (yrs)**	**Sex**	**Normal**	**(%)**	**Overweight**	**(%)**	**Obese**	**(%)**	**Unknown**	**(%)**	**Total**	**(%)**

**15 – 25**	Male	10	23%	7	14%	2	.02%	0	0%	19	10%
	Female	7	16%	5	10%	9	12%	0	0%	21	12%
	
	**Total**	17	39.5%	12	24%	11	14%	0	0%	40	22%

**26 – 45**	Male	5	12%	13	26%	18	23%	4	33%	40	22%
	Female	14	33%	9	18%	18	23%	5	42%	46%	25%

	**Total**	19	44%	22	44%	36	47%	9	75%	86	47%

**> 45**	Male	4	9%	6	12%	13	17%	1	8%	24	13%
	Female	3	7%	10	20%	17	22%	2	17%	32	18%
	
	**Total**	7	16%	16	32%	30	39%	3	25%	56	31%

	**TOTAL**	**43**	**100%**	**50**	**100%**	**77**	**100%**	**12**	**100%**	**182**	**100%**

**Note: **BMI = Weight (kg) divided by square of height (m)

#### Self-Report of LBP within the last seven days

The prevalence of all LBP (i.e. including all levels of pain) within the last seven days was 72% (95% CI: 63%–80%) and all LBP lasting seven weeks or longer was 34 % (95% CI: 27%–40%).

#### Previous history of LBP

Previous history of LBP was present in 34% (95% CI: 27%–40%) of respondents. A previous history of LBP is known to predispose individuals to recurrent episodes of back pain [31].

### Other modifiable risk factors for LBP

#### Smoking

Smoking was highly prevalent 46% (95% CI: 38%–53%) in the community, with equal numbers of males and females smoking. Thirty eight per cent (95% CI: 31%–45%) of people smoked between 10–20 cigarettes daily and 8% (95% CI: .04%–11%) smoked more than 20 cigarettes per day. This is consistent with the 2001 National Health Survey (NHS), which found that 51% of Indigenous people aged 18 years or older were current smokers, compared with 24% of non-Indigenous people [32].

#### Physical inactivity

Sixteen percent (95%CI: 10%–21%) of participants spent no time actively exercising and 35.9% (95% CI: 26%–45%) exercised less than 30 minutes per week. There are no other detailed data available on the levels of physical activity among Indigenous people. However, the 2001 NHS reported that 43% of Indigenous people aged 18 years or older living in non-remote areas were sedentary, compared with 30% of non-Indigenous people [32].

#### Psychosocial stress

For those reporting LBP 72% (CI: 65%–78%), the most commonly reported traumatic events included sporting injuries 26.5% (95% CI: 20%–38%), motor vehicle accidents 18% (95% CI: 12%–23%) and work-related trauma 17.5% (95% CI: 12%–22%). There was, however, no association between LBP and physical trauma.

#### Physical trauma

For those reporting LBP (66.1% CI: 54%–68%), the most commonly reported traumatic events included sporting injuries 26.5% (95% CI: 20%–38%), motor vehicle accidents 18% (95% CI: 12%–23%) and work-related trauma 17.5% (95% CI: 12%–22%). There was, however, no association between LBP and physical trauma.

#### Occupational risk factors

**Figure 1:**
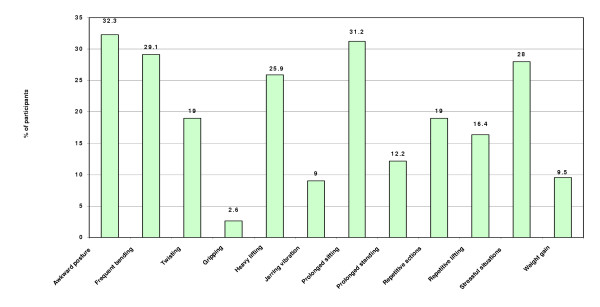
Figure 1

Figure 1, Modifiable occupational risk factors for musculo-skeletal conditions details reported occupational risk factors for LBP. Common risk factors were adopting awkward postures at work 32% (95% CI: 25%–39%), frequent bending and twisting 29% (95%: CI: 22%–35%) and heavy lifting 26% (95% CI: 20% – 32%). However, there was no association between LBP and occupational risk factors.

### Factors associated with reported LBP

Even though a trend was evident, no statistical association between LBP and the lifestyle factors detailed above. However, more participants reporting high levels of LBP were overweight or obese and obesity was statistically associated with self-reported strain causing reported LBP (χ^2 ^= 9.02, df = 2 10, p = 0.01). While sporting injuries were not statistically associated with report of LBP in particular, participants reporting sporting injuries experienced between two and four musculo-skeletal conditions (χ^2 ^= 7.90, df = 2, p = 0.02).

## Discussion

The 72% seven day prevalence of LBP found in the Kempsey survey is greater than similar prevalence levels reported in other rural Indigenous Communities [1,2,33,34]. In their study, Honeyman and Jacobs [2] reported a 1-day LBP prevalence for the majority of community members, 68% (95% CI: 61%–74%). The majority of participants in the Kempsey survey also experienced their presenting LBP for seven weeks or more. Thus according to accepted definitions of chronicity [35], the majority of Indigenous people in this Community were suffering from chronic pain and were therefore, likely to be at greater risk of enduring prolonged disability [31]. Thirty-four percent of participants also reported a previous history of LBP, which was likely to predispose them to recurrent, future episodes [31]. Furthermore, trauma particularly that incurred in sporting injuries was associated with multiple musculo-skeletal conditions. Past studies have reported that Indigenous people are more likely to experience transport accidents, intentional self-harm and assault than other Australians with rates approximating three times those of the rest of the Australian population [32].

The findings in this study of higher levels of smoking, physical inactivity and obesity are consistent with those reported by other studies of Indigenous Australians [9]. Though many of the modifiable risk factors known to be associated with LBP were highly prevalent in this study, none of these were statistically associated with LBP. One explanation for this finding is that the size of the sample, though sufficiently large to demonstrate comparability with ABS findings for demographic categories, may not have been sufficiently large to achieve the statistical power to detect any association between LBP and associated study factors.

Obesity and physical inactivity are the two most important modifiable factors contributing to the development of type 2 diabetes mellitus. These factors were highly prevalent in the community with 26% of subjects overweight, 45% obese and 16% spending no time actively exercising plus a further 35.9% exercising less than 30 minutes per day. Exercising was assessed by self-report according to total time spent exercising ranging from 'No time' to 'More than 10 hours per week'. Obesity in this study was associated with self-reported low back strain. The prevalence of obesity in this community is of concern, first because obesity is an independent predictor of back pain [36], but more importantly as obesity has a global health impact.

Health providers including chiropractors and osteopaths commonly counsel LBP sufferers to lose weight to unload their spines. Weight loss also offers other musculo-skeletal benefits. Females with a BMI of over 25 kg/m2, can, by losing 5 kg (2 BMI units) reduce future onset of knee osteoarthritis by 50% and males by 25% [37]. Obesity has also been associated with a higher prevalence of work limitations, hypertension, dyslipidemia, type 2 diabetes and the metabolic syndrome in adults of working age [38]. Furthermore, Australia-wide some 50% of cases of type 2 diabetes are asymptomatic, undiagnosed and persons subclinically undergo progressive macro and micro-vascular changes [39]. The current findings suggest that screening this population group for evidence of glucose intolerance when reviewing musculo-skeletal conditions such as LBP may be valuable.

Of those reporting LBP, 72% of participants (CI: 65%–78%) were frequently exposed to "stressful situations" in their occupation. However, psychosocial stress outside of the work place was not measured given the cultural sensitivity of this factor according to the CAG. Psychosocial stress in general is a strong predictor of LBP [40,41]. If conducted in a culturally appropriate manner, future studies assessing LBP in Indigenous Communities should ideally attempt to also measure psychosocial stress as a potential contributing study factor.

Another concurrent health hazard is the high prevalence of cigarette smoking. In addition to the well documented risks of smoking it has been found that compared with matched groups of non-smokers, chronic cigarette smokers are more likely to be insulin resistant, hyperinsulinemic, and dyslipidemic [39].

Exercise is the most common method of treating LBP in Australia [42]. In addition it may be the single most important lifestyle factor for both preventing and reversing insulin resistance, particularly among obese individuals [12,13]. This suggests a good case for concentrating on general exercise health promotion for Indigenous communities.

Lifestyle interventions incorporated into a culturally sensitive health promotion program could potentially benefit the health and modify the morbidity and mortality of this population group. These results suggest an opportunity to review and address risk factors associated with LBP along with more serious diseases affecting Indigenous people. Addressing modifiable risk factors associated with LBP, such as smoking, physical inactivity, and obesity could significantly contribute to the management of co-morbidities including diabetes and heart disease which so commonly affect Indigenous Australians.

An understanding of the modifiable risk factors for LBP identified in this paper also formed the basis for a culturally acceptable musculo-skeletal intervention designed to address the high prevalence of LBP. This involved using a pilot training program for Aboriginal Health Workers (AHWs). The intervention was designed to promote the musculo-skeletal and general health of Indigenous people living in this rural community [12]. Culturally sensitive approaches to managing musculoskeletal conditions have been successfully implemented in other Indigenous Communities [43].

The Community Oriented Program for the Control of the Rheumatic Diseases (COPCORD) represents the largest, ongoing collaborative attempt to measure the prevalence of musculo-skeletal conditions and risk factors in rural populations throughout the world [43]. COPCORD has also developed implemented and evaluated culturally sensitive approaches for managing these conditions and their associated risk factors through community-based initiatives with applicability in other Indigenous Communities.

We propose that any future musculo-skeletal study or intervention in an Indigenous community be accompanied by a review of the modifiable risk factors associated with LBP and counselling about those factors. This may have a beneficial effect on the overall well being of indigenous communities. Further research should test such a program for efficacy and effectiveness.

## Conclusion

The disturbingly high prevalence of LBP experienced in this community necessitates a serious response. Managing LBP through health services and addressing the modifiable risk factors through culturally sensitive, health promotion programs will be an important step in addressing the high burden of illness imposed by LBP and other more serious conditions suffered in this community.

## Competing interests

Dr. Bruce Walker is Editor-in-Chief of *Chiropractic & Osteopathy*.
